# Integrated Management Strategies for Wood Infested by *Hylurgus ligniperda* F. (Coleoptera: Curculionidae: Scolytinae)

**DOI:** 10.3390/insects16111154

**Published:** 2025-11-11

**Authors:** Huanwen Chen, Xiaowei Chen, Dan Xie, Qingshan Yang, Fang Niu, Defu Chi, Jia Yu

**Affiliations:** 1 College of Forestry, Northeast Forestry University, Harbin 150040, China; chenhuanwen2020@126.com (H.C.); hcoeng5@163.com (X.C.); xiedan0807@126.com (D.X.); 2 Forestry Bureau of Muchuan County, Leshan 614500, China; gjjhfhjk@163.com; 3 College of Traffic and Transportation, Harbin Railway Technical College, Harbin 150060, China; niufang890526@126.com

**Keywords:** *Hylurgus ligniperda*, fumigation, sulfuryl fluoride, aluminum phosphide, thermal treatment, beta-cypermethrin

## Abstract

The red-haired bark beetle (RHB), *Hylurgus ligniperda* (Fabricius, 1787) (Coleoptera: Curculionidae: Scolytinae), is a globally distributed quarantine pest, making effective management of infested wood essential. This study developed an integrated control system to achieve closed-loop management under various environmental and wood conditions. RHB eggs were the most fumigation-tolerant stage. Although sulfuryl fluoride (SF) showed higher potency, aluminum phosphide (AP) provided deeper penetration into wood (AP: 29.5% vs. SF: 12.6%). Both fumigants effectively reached all logs in the stacks. Fumigation efficacy was highest at moderate temperatures (18–22 °C) and lower wood moisture levels. In addition to chemical methods, thermal treatments were evaluated: heating to 60–65 °C achieved complete mortality, while effective freezing control required temperatures of −30 °C. For bark and chip debris generated during processing, beta-cypermethrin fumigation effectively eliminated residual pests. Our results confirm that both AP and SF are suitable for disinfecting infested *Pinus thunbergii* wood, that extreme temperatures can reduce dispersal risks, and that beta-cypermethrin is effective in the downgraded utilization of wood byproducts. This work provides a comprehensive and practical framework for controlling the spread of this invasive pest.

## Simple Summary

The red-haired bark beetle is a destructive forest pest that is spreading globally. A major challenge in its management is the safe disposal of infested trees to prevent further dispersal. Our study demonstrates that sulfuryl fluoride is highly effective, requiring only a quarter of the dosage of aluminum phosphide to achieve mortality, even against the fumigation-resistant egg stage. However, aluminum phosphide exhibited superior penetration depth in wood, making it more effective against deeply concealed beetles. Fumigation efficacy was highest at moderate temperatures and under drier wood conditions. We also showed that exposing the wood to high heat (above 60–65 °C) or extreme cold (below −30 °C) killed all beetle life stages. For bark debris generated during wood processing, beta-cypermethrin fumigation was highly effective against residual pests. Our results provide a comprehensive and practical strategy for managing infested wood, helping to protect global forests from this invasive species.

## 1. Introduction

The expansion of global trade has inadvertently accelerated the spread of invasive bark beetles, posing a significant threat to forest ecosystems and resulting in substantial economic losses on a global scale [1,2]. A prominent example of a successful invader is the red-haired bark beetle (RHB), *Hylurgus ligniperda* (Fabricius) (Coleoptera: Curculionidae), native to the western Palearctic region [3,4]. RHB is noted for its broad host range, high reproductive rate, and rapid dispersal capability [5,6], traits that have facilitated its colonization in numerous non-native regions worldwide [7,8]. RHB primarily infests recently felled logs and stumps [9]. RHB can also act in concert with other pests to accelerate tree decline. Under high population density, it is capable of attacking healthy trees and seedlings [4,10]. The symbiotic fungi vectored by the RHB, primarily ophiostomatoid fungi, trigger blue-stain in the wood, ultimately causing a substantial loss in its economic value [11,12,13]. The widespread presence of RHB in coastal forests and its potential for outbreaks constitute a serious and growing threat to the stability of these ecosystems.

Effective integrated management of the RHB includes the critical step of disrupting its life cycle within infested wood. This pest exhibits overlapping generations, completes 1–3 generations annually in invaded areas, and frequently produces sister broods, resulting in rapid population growth [6,14]. Therefore, the timely and effective treatment or disposal of infested wood represents a core measure of Integrated Pest Management (IPM), aimed at directly eliminating eggs, larvae, pupae, and adults [15]. For imported or exported wood showing signs of RHB infestation, it is crucial to implement phytosanitary treatments to prevent its colonization and dispersal in and around port areas.

Methyl bromide fumigation has been a common phytosanitary treatment for wood, but its role as an ozone-depleting substance [16] has led to its global phase-out [17,18]. Several alternative fumigants have emerged as promising replacements, demonstrating efficacy against a range of forest pests [19,20,21,22]. In addition, non-chemical methods such as wood chipping, heat treatment, and cold treatment can also be employed to eliminate pests and pathogens [23,24,25]. The global success of RHB as an invader highlights inadequacies in current practices for managing infested wood. The difficulties likely stem from the RHB’s concealed habits and the thick bark of its hosts, which hinder effective penetration of fumigation, as well as an insufficient systematic evaluation of control methods, preventing strategy optimization.

However, the efficacy of current infested wood management still faces multiple bottlenecks. Firstly, the performance of alternative fumigants is significantly influenced by environmental factors [26,27,28]. There is a lack of systematic research on fumigation dynamics under varying temperature and humidity conditions, hindering the development of precise application protocols. Secondly, while physical heat treatment is an effective method for eliminating propagable insect sources within infested wood [29,30], the precise temperature-time thresholds required to completely inactivate all cryptic life stages inside large-diameter logs remain unclear. Furthermore, by-products from wood processing, such as bark debris, represent a frequently overlooked pathway for pest dispersal [31]. There is a pressing need to develop targeted treatment strategies to achieve closed-loop management.

Based on the invasion of RHB in Yantai, Shandong Province, China, this study systematically evaluated the efficacy of fumigation and temperature-based treatments in disinfecting infested wood. The research objectives were threefold: (1) to determine the toxicity of Aluminum phosphide (AP) and Sulfuryl fluoride (SF) to RHB all developmental stages and their wood penetration, and to clarify the effects of temperature and humidity on fumigation; (2) to establish the critical temperature-time thresholds required to achieve complete mortality of all RHB developmental stages within infested wood using both high and low temperatures; and (3) to optimize fumigation and heat treatment protocols for the bark debris generated from processing damaged wood, addressing this potential dispersal pathway. The findings provide key technical parameters for controlling RHB populations in infested areas and offer practical solutions for the safe handling and cross-border management of RHB-infested wood globally.

## 2. Materials and Methods

### 2.1. Insect Samples and Infested Wood

Larvae and adults of RHB, along with infested wood, were collected from a coastal protection forest in Muping District, Yantai City, Shandong Province, China (37°27′36″ N, 121°51′25″ E). Adult beetles were captured using lure-baited traps, with the attractant prepared according to the method of Gu et al. [32]. Larvae were obtained from stumps exhibiting clear frass extrusion, carefully dissected, and individuals of comparable size were selected for subsequent fumigation assays. Due to the short pupal stage and low natural abundance of RHB, we used field-collected mature larvae and reared them on an artificial diet until pupation. The pupae could then be clearly observed at the bottom of the Petri dish [33]; uniformly developed pupae were then used. Eggs were laid by field-collected adults under laboratory conditions, following the egg collection procedure described by Clare [7]. Infested wood showing signs of RHB damage was harvested from trunks and roots and cut into segments of different sizes for subsequent experiments: 30 cm long pieces were used for fumigation assays, while logs measuring 50, 100, and 150 cm in length were assembled into stacks for wood-penetration tests. The RHB-infested wood logs used in the fumigation trials had diameters ranging from 28 to 37 cm.

### 2.2. Toxicity Testing of Fumigants Against RHB

#### 2.2.1. Susceptibility of Different Developmental Stages to Fumigants

Live individuals from all RHB developmental stages (eggs, larvae, pupae, and adults) were placed separately in 90-mm-diameter glass Petri dishes, each containing 10 g of artificial diet prepared according to Romo [33]. The insects were positioned on the diet surface to ensure full contact with the fumigant. Each dish contained 50 eggs, 30 larvae, 30 pupae, or 30 adults. The uncovered Petri dishes were then wrapped with gauze. The dishes were transferred into sealed polyethylene (PE) containers for fumigation. All fumigation trials were carried out in an incubator maintained at 20 °C. After treatment, mortality counts for each developmental stage were recorded.

During fumigation, SF was applied at eight evenly spaced dosage levels (1, 3, 5, 7, 9, 11, 13, 15 mg·h·L^−1^) with a uniform exposure time of 8 h. AP was applied at ten evenly spaced dosage levels (1.2, 2.4, 3.6, 4.8, 6, 7.2, 8.4, 9.6, 10.8, 12 mg·h·L^−1^) with a uniform exposure time of 30 h. For each RHB life stage, 150 individuals were used as replicates (comprising 3 Petri dishes for eggs, and 5 Petri dishes each for larvae, pupae, and adults).

#### 2.2.2. Assessment of Fumigant Penetration Ability in Wood and Wood Stacks

Rectangular specimens (5 cm × 10 cm × 10 cm) were prepared from *P. thunbergii* wood blocks. A cavity measuring 2.5 cm × 3 cm × 1.25 cm was created in the center of one 10 cm × 10 cm face. Adult RHB were introduced into this cavity, which was then covered with another identically prepared wood block featuring a polished surface. External pressure was applied manually using a twisting motion to ensure tight contact between the two sanded smooth wood surfaces, thereby forming an effectively sealed chamber for fumigation. In our preliminary experiments, the seal integrity of the wood blocks was verified using a soap bubble test and was found to fully meet the experimental requirements. After fumigation, insect mortality inside the chamber was recorded. Wood blocks without sealed cavities served as controls for evaluating the penetration ability of fumigant molecules. All wood blocks were wrapped in gauze to prevent insect escape. This experiment was conducted in mid-May 2023 at temperatures ranging from 18.5 to 25.5 °C.

The fumigant penetration rate was calculated as follows:

Fumigant penetration rate (%) = (LCT_99_ in exposed fumigation/LCT_99_ in sealed fumigation) × 100%, where LCT_99_ is the lethal concentration-time product for 99% mortality.

The test for fumigant penetration into wood stacks was conducted inside sealed tents. A wood stack with a side length of 3 m was constructed, and adult RHB were placed into open PE boxes (50 beetles per box). Each box contained a gauze-wrapped, defatted cotton ball to maintain humidity. The open boxes were covered and sealed with gauze to prevent insect escape. These boxes were then positioned within the wood stack at depths of 0.5, 1.0, and 1.5 m for fumigation. The experiment was conducted in mid-May 2023 at temperatures between 18.5 and 25.5 °C.

### 2.3. Fumigation Treatment of RHB in Infested Wood

#### 2.3.1. Effect of Temperature on Fumigation Efficacy

To examine the effect of temperature on fumigation efficacy, the wood moisture content was maintained between 20.6% and 24.8%. The experiments were carried out in mid-April, mid-May, and mid-June 2023 to take advantage of natural temperature fluctuations, which yielded three corresponding test temperature ranges: 11.5–15.4 °C, 18.5–25.5 °C, and 26.5–29.5 °C. A loading ratio of 50% was used for the infested wood (i.e., a 1:2 ratio of total wood segment volume to fumigation container volume). The trial included five application rates, each tested over 4–5 different exposure durations. SF was applied at five evenly spaced dosage levels (10, 20, 30, 40, 50 mg·h·L^−1^) with exposure times of 24, 36, 48, 54, 56, 60 h, respectively. AP was applied at five evenly spaced dosage levels (6, 12, 18, 24, 30 mg·h·L^−1^) with exposure times of 24, 72, 120, 168 h, respectively.

#### 2.3.2. Effect of Wood Moisture Content on Fumigation Efficacy

This study evaluated the effects of wood moisture content on fumigation efficacy under natural ambient conditions. Fumigation trials were conducted outdoors to simulate real-world scenarios, with recorded temperatures ranging from 18.5 to 25.5 °C, representing the minimum and maximum values observed during the experiments. Wood moisture content was determined by analyzing 2-cm-thick sections from infested logs, which were subsequently categorized into three distinct moisture ranges for testing: 21.34–25.5%, 31.6–33.6%, and 45.76–48.76%, to systematically assess the influence of moisture variation on treatment outcomes. Prior to fumigation, the wood segment volume was measured and a 50% loading ratio was maintained. SF was applied at eleven spaced dosage levels (5, 10, 15, 20, 25, 30, 35, 40, 45, 60, 65 mg·h·L^−1^) with exposure times of 48 h. AP was applied at eight spaced dosage levels (6, 12, 18, 24, 30, 36, 42, 48 mg·h·L^−1^) with exposure times of 72 h.

#### 2.3.3. High- and Low-Temperature Treatment of RHB-Infested Wood

For the high- and low-temperature treatment experiments, the moisture content of the infested wood was maintained at 30–34% (The specific screening procedure for wood moisture content is described in Section 2.3.2). High-temperature treatments were tested at 45 °C, 50 °C, 55 °C, 60 °C, and 65 °C; low-temperature treatments were evaluated at −15 °C, −20 °C, −25 °C, −30 °C, and −35 °C. The exposure durations tested varied with temperature. At low temperature, nine exposure durations were applied: 5, 10, 15, 16, 20, 25, 30, 35, and 40 h. At high temperature, ten exposure durations were applied: 2, 4, 5, 10, 15, 20, 25, 30, 35, and 40 h. After treatment, the wood segments were dissected and insect mortality was recorded. The temperature sensitivity of RHB was assessed using lethal exposure time (LET) values. During actual fumigation of infested wood, adults and larvae represented the majority of the population, whereas eggs and pupae were rarely encountered. Therefore, RHB mortality was recorded collectively across all developmental stages. As RHB does not infest deep xylem, heartwood temperature recording is unnecessary, and only the phloem temperature requires monitoring.

### 2.4. Treatment of Bark and Wood Chips During Downgrading of Infested Wood

During the chipping of severely infested wood with low utilization value, bark detachment frequently occurs, creating a risk of secondary pest dispersal, as multiple developmental stages of RHB can survive in the resulting bark debris and wood chips. Due to the high cost of temperature-based treatment systems, this study evaluated insecticidal fumigation as a more feasible control option. The tested insecticides included dichlorvos (Emulsifiable Concentrate, EC), lime sulfur (Soluble Concentrate, SL), and beta-cypermethrin (Fumigant, FU). Throughout the 48-h fumigation period, all RHB individuals within the bark debris were directly exposed to the insecticides. Adult and larval mortality was assessed by examining all individuals, whereas egg mortality was evaluated using approximately 1 kg samples of dislodged frass. Due to the high mortality of pupae caused by mechanical effects (friction between detached bark and pupae), they were not included in the statistical analysis.

Beta-cypermethrin fumigant tablets were applied at 20 g, dichlorvos at 1.25 mL, and lime sulfur at 3.45 mL. Each insecticide was placed in a round iron container (10 mm in diameter) to fumigate a total of 10 kg of bark and wood chip debris. The bark to be fumigated was placed on wire mesh racks to ensure unimpeded fumigant circulation and full contact with the beetles. The bark layer thickness was kept below 5 cm.

In practice, it is imperative to maintain the sample loading rate below 50% and to use a sealed container with a structure that facilitates gas circulation. In this study, a 80 L container was used for a 10 kg sample.

### 2.5. Chemicals and Apparatus

SF (purity 99%, Hunan LikeDe Energy Technology Co., Ltd., Changsha, China), AP tablets (56% purity, Jining Yimin Chemical Co., Ltd., Jining, China), dichlorvos (80% EC, Planck Biochemical Industry Co., Ltd., Martinsried, Germany), lime sulfur (29% SL, Hebei Shuangji Chemical Co., Ltd., Xinji, China), and beta-cypermethrin (5% FU, Shanxi Aosainuo Biotechnology Co., Ltd., Taiyuan, China) were used as test chemicals. Fumigation was conducted in 50 L high-density polyethylene barrels, each equipped with a small internal fan to ensure uniform gas distribution and a bluetooth digital thermometer for real-time temperature monitoring. Thermal treatments were performed using a constant-temperature forced-air drying oven (Shanghai Yiheng Scientific Instrument Co., Ltd., Shanghai, China), while cold treatments utilized a temperature-controlled freezer (AUX Group Co., Ltd., Ningbo, China). An artificial climate chamber (Shaoxing Shangcheng Instrument Manufacturing Co., Ltd., Shaoxing, China) was used for maintaining specific environmental conditions.

### 2.6. Mortality Assessment of Test RHB

Following fumigation, larvae, pupae and adult beetles were held in diet-containing Petri dishes at 25 °C and 75% relative humidity for 72 h. Individuals showing no movement in response to gentle physical stimulation under a dissection microscope were recorded as dead. Eggs were incubated on filter paper moistened with sterile phosphate-buffered saline (PBS) for 7 days; those that failed to hatch were considered non-viable. Due to the pronounced death-feigning behavior exhibited by RHB, this assessment method was consistently applied across all relevant experiments to ensure the accuracy of the experimental results.

### 2.7. Statistical Analysis

The lethal concentration-time (LCT) values for fumigation experiments and the lethal exposure time (LET) values for temperature-stress experiments were calculated using probit analysis. Probit regression models were fitted to the dose–response data (for LCT) and the time-mortality data (for LET) using the glm function in R with a probit link function. The LCT_50_ and LCT_99_ values represent the concentration-time products required for 50% and 99% mortality, respectively. Correspondingly, the LET_99_ values represent the exposure time required to achieve 99% mortality at a specific high or low temperature [34]. The LCT50 and LET50 values and their 95% confidence intervals (95% CI) for each experimental group were calculated based on probit analysis. We employed the Pearson chi-square goodness-of-fit test to assess model fit. The statistical significance of differences between LCT50 and LET50 values from different groups was assessed by examining the overlap of their 95% confidence intervals; as a general rule, non-overlapping confidence intervals indicate a significant difference at the α = 0.05 level.

Generalized Linear Mixed Models (GLMMs) were employed to evaluate the lethal effects of different insecticide treatments on various developmental stages of RHB. Separate models were constructed for each treatment duration (24 h, 48 h, and 72 h), with the bivariate response variable of dead and alive counts as the dependent variable. A binomial distribution with logit link function was used to model the mortality proportion data. Fixed effects in the models included insecticide type (4 levels: control, dichlorvos, lime sulfur, beta-cypermethrin), developmental stage (3 levels: egg, larva, adult), and their two-way interaction. Experimental replication (3 replicates) was incorporated as a random effect to account for random variation arising from repeated measurements. Model fitting was implemented using the lme4 package, with parameter estimation conducted via maximum likelihood Laplace approximation. Likelihood ratio tests were performed to compare models with and without interaction terms to determine the necessity of interaction effects. For significant fixed effects, least-squares means were estimated using the emmeans package, followed by post hoc multiple comparisons with Tukey’s method for *p*-value adjustment to control the family-wise error rate. Model diagnostics were conducted to check for singular fits and overdispersion, ensuring that model assumptions were adequately met. All statistical analyses were performed in R version 4.5.1.

## 3. Results

### 3.1. Fumigation Toxicity of AP and SF Against Various Developmental Stages of RHB

To evaluate the efficacy of AP and SF against all developmental stages of RHB, tests were carried out simultaneous fumigation experiments in 50 L sealed container (Table 1). The results indicated that SF exhibited superior efficacy when insects were fully exposed to the fumigant atmosphere. For each developmental stage, the lethal concentration-time (LCT_50_ and LCT_99_) values for AP were consistently higher than those for SF. Specifically, the LCT_50_ and LCT_99_ values of AP were 2.8- and 3.9-fold higher than those of SF in eggs, 3.1- and 4.7-fold higher in larvae, 2.9- and 4.4-fold higher in pupae, and 2.6- and 2.8-fold higher in adults, respectively. Overall, adults showed the lowest tolerance to both fumigants, whereas eggs exhibited the highest tolerance. At 20 °C, the LCT_50_ and LCT_99_ values for AP were 118.7 and 808.0 mg·h·L^−1^ for eggs, and 57.4 and 368.4 mg·h·L^−1^ for adults, respectively. In contrast, the corresponding values for SF were 41.8 and 204.7 mg·h·L^−1^ for eggs, and 22.2 and 132.4 mg·h·L^−1^ for adults. For the same developmental stage, a significant difference (*p* < 0.05) was observed between the LCT50 values of AP and SF, with the 95% confidence interval of the former being entirely higher than that of the latter.

### 3.2. Penetration of AP and SF into P. thunbergii Wood

Comparative fumigation experiments with AP and SF were performed on adult RHB in *P. thunbergii* wood blocks under both opened and sealed conditions (Table 2). The results showed that the LCT_99_ values for AP were consistently higher than those for SF across all conditions. Under opened conditions, the LCT_99_ for AP was 279.4 mg·h·L^−1^, which was 3.4 times that of SF. Under sealed conditions, the LCT_99_ for AP increased to 948.7 mg·h·L^−1^, 1.6 times that of SF. Moreover, a notable difference was observed in the wood penetration ability between the two fumigants. The penetration rate of AP through the *P*. *thunbergii* wood blocks reached 29.5%, compared to only 12.6% for SF.

In fumigation trials on infested *P*. *thunbergii* wood stacks (Table 3), both AP and sulfuryl fluoride effectively penetrated 3 m^3^ wood piles and successfully eliminated adult RHB inside. Penetration efficiency, however, varied with stack depth: the LCT_99_ value for AP rose progressively with depth, while that of sulfuryl fluoride remained stable up to 1 m and increased only slightly (by 40%) at 1.5 m. With the exception of the significant difference in LCT_99_ between 0.5 m and 1.5 m depths in the AP treatment (as indicated by non-overlapping 95% confidence intervals) (*p* < 0.05), no significant difference was observed in the overall penetrability between the two fumigants within the wood stacks.

### 3.3. Influence of Fumigation Conditions on AP and SF Efficacy Against RHB in Infested Wood

Infested wood logs were fumigated within sealed tents to evaluate the insecticidal activity of the two fumigants against RHB. Under conditions of fixed wood moisture content, fumigation efficacy was highest within the temperature range of 18.5–25.5 °C (Table 4). Within this optimal range, the LCT_99_ values for both AP and SF reached their minima, at 6421.7 mg·h·L^−1^ and 5101.0 mg·h·L^−1^, respectively. Under these conditions, the LCT_99_ for AP was 1.3 times that of SF. The 95% confidence intervals of their LCT_99_ values show substantial overlap, indicating no statistically significant difference. Nevertheless, maintaining temperatures within or near the 18.5–25.5 °C range may still enhance fumigation efficacy against RHB-infested wood.

With ambient temperature maintained at 18.5–25.5 °C, the effect of wood moisture content on fumigation efficacy was examined (Table 5). The results indicated that lower wood moisture content correlated with higher insecticidal efficacy for both fumigants. When the moisture content was between 21.3–25.5%, the LCT_99_ values for both AP and SF were the lowest, recorded at 6264.6 mg·h·L^−1^ and 6740.3 mg·h·L^−1^, respectively. In this scenario, the LCT_99_ for AP was 0.96 times that of SF. The differences in LCT_99_ values of the fumigant required at different moisture content levels were not statistically significant.

### 3.4. Heating and Freezing Treatment Conditions for RHB in Infested Wood

Infested wood samples were placed in high- and low-temperature treatment equipment, and mortality rates of RHB were recorded after fixed exposure periods (Table 6). Under high-temperature treatment (45–65 °C), the difference in lethal exposure time decreased significantly with each 5 °C temperature increase (ΔLET_99_), dropping successively from 296.3 h to 20.6 h, 9.9 h, and 0.7 h, indicating a sharp reduction in additional exposure time required at higher temperatures. Similarly, under low-temperature treatment (−15 to −35 °C), the ΔLET_99_ also decreased markedly with each 5 °C decrease, declining from 68,916.6 h to 294.8 h, 174.4 h, and 5.7 h. Overall, RHB demonstrated greater sensitivity to high temperatures. Under extreme conditions of 65 °C and −35 °C, the cumulative LET_99_ values were 6.3 h and 26.4 h, respectively, further confirming the higher efficiency of thermal lethality at elevated temperatures.

### 3.5. Management Strategies for Bark and Chip Byproducts from Processing RHB-Infested Wood

**Figure 1:**
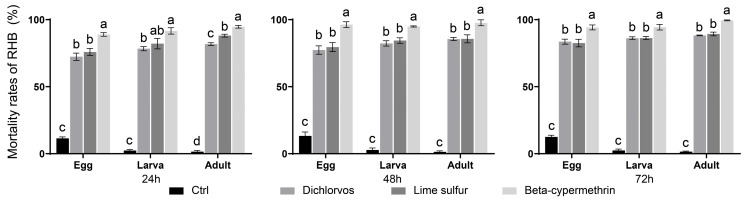
Efficacy of three fumigant insecticides against RHB at different developmental stages in bark stripped from infested wood. Different letters indicate significant differences in insecticidal efficacy among the various insecticides within the same developmental stage (*p* < 0.05).

Fumigation experiments were conducted using three insecticides—dichlorvos EC, lime sulfur SL, and beta-cypermethrin FU—against RHB at different developmental stages in the bark of low-value, infested wood (Figure 1). GLMMs consistently revealed significant effects of insecticide and developmental stage on insect mortality across different treatment durations. At the 24-h treatment, a significant insecticide × developmental stage interaction was observed (χ^2^ (6) = 83.28, *p* < 0.001), along with highly significant main effects of both insecticide (χ^2^ (3) = 1285.60, *p* < 0.001) and developmental stage (χ^2^ (2) = 54.54, *p* < 0.001). Similarly, the 48-h treatment also demonstrated significant interaction (χ^2^ (6) = 66.80, *p* < 0.001), insecticide main effect (χ^2^ (3) = 1610.40, *p* < 0.001), and developmental stage main effect (χ^2^ (2) = 56.21, *p* < 0.001). At the 72-h treatment, significant interaction (χ^2^ (6) = 101.91, *p* < 0.001) was likewise detected, accompanied by significant main effects of insecticide (χ^2^ (3) = 1281.16, *p* < 0.001) and developmental stage (χ^2^ (2) = 55.03, *p* < 0.001), indicating that the tested insecticides exhibited distinct efficacy patterns against different developmental stages, and these differential patterns remained consistent across the various treatment durations. The results revealed complex interactions between insecticide type and developmental stage. In general, adults tended to exhibit the highest susceptibility among the mobile stages (larvae, pupae, adults), particularly at earlier exposure times (e.g., 24 h for dichlorvos). However, this pattern was not universal, as no significant differences in mortality were found among larvae, pupae, and adults when treated with beta-cypermethrin. Eggs consistently showed the lowest susceptibility across all insecticides and time points. During mortality assessments, surviving adults were primarily found in areas with thicker phloem at the junctions of trunks and lateral branches. Subsequent Tukey HSD post hoc tests demonstrated that beta-cypermethrin resulted in significantly higher mortality rates than both dichlorvos and lime sulfur at all three time points (*p* < 0.05). After 72 h of fumigation, mean mortality rates with beta-cypermethrin reached 94.2% for eggs, 94.4% for larvae, and 99.5% for adults, highlighting its strong potential for practical management of RHB-infested, low-value wood in affected regions.

## 4. Discussion

The ongoing global dispersal and establishment of RHB [35] highlights critical gaps in existing quarantine and control measures [36]. This invasive pest is frequently spread over long distances through the international trade of infested wood [37,38,39]. We conducted a systematic evaluation of two main alternative fumigants and extreme temperature treatments for their efficacy in controlling RHB in infested wood. Our findings indicate that the choice between SF and AP represents a trade-off between insecticidal toxicity and wood penetration capacity. Furthermore, we precisely determined the temperature-time thresholds for lethal exposure under both high- and low-temperature conditions and validated a targeted management strategy for processing byproducts. These results provide comprehensive and reliable empirical support for developing local population reduction protocols and enhancing the phytosanitary management framework for RHB-infested wood.

Both AP and SF exhibited significant fumigation toxicity, though with distinct modes of action [40]. In this study, eggs and pupae of RHB showed higher tolerance to both fumigants, whereas adults were the most susceptible. Compared to active larvae and adults, eggs and pupae exhibit significantly lower respiratory metabolic rates. Since fumigants primarily enter the insect body through the respiratory system, this reduced metabolic rate may directly lead to decreased uptake rate and accumulation of the fumigant. This pattern is consistent with fumigation responses reported for many other pests [41,42]. The LCT_99_ values required for SF and AP against RHB fell within the intermediate range compared to those reported for other Coleoptera [43,44]. Notably, AP demonstrated significantly greater wood penetration (29.5%) than SF (12.6%), which can be attributed to the distinct physicochemical properties of their active gases. Compared to SF, phosphine (PH_3_) released from AP has a smaller molecular size and lower adsorption tendency (National Institute of Standards and Technology), potentially facilitating more effective diffusion through the complex pore structure and bark of *P*. *thunbergii*. While SF is suitable for rapid knockdown of exposed pests, AP is indispensable for targeting deeply concealed populations within thick-barked wood. It is worth noting that within the temperature range tested, fumigation efficacy did not increase continuously with rising temperature. Higher temperatures may promote the adsorption of fumigant gases onto wood, thereby reducing the effective gas concentration in the environment. Similarly, wood moisture content played a critical role: higher moisture levels reduced fumigation efficiency, likely by interfering with fumigant penetration through the wood. These insights are essential for developing predictive models and optimizing practical treatment protocols. Although the probit model showed a significant lack of fit (as indicated by the goodness-of-fit test), this does not undermine the practical significance of our findings. This statistical limitation likely stems from the high efficacy of the fumigants, which caused a rapid transition to 100% mortality, a desirable trait in pest control operations. Additionally, to ensure the experimental data provides practical guidance, we continued to increase the fumigant dosage and exposure time even after 100% mortality of RHB was achieved, which may also have contributed to the poor model fit. For practical purposes, the empirical data unequivocally identify the concentrations required for complete control, providing a reliable and actionable guide for field application.

Extreme temperature treatments also serve as highly effective methods for managing infested wood [45,46,47]. Our experiments revealed that the LET_99_ values under high-temperature conditions were significantly lower than those under freezing conditions, demonstrating that RHB is more vulnerable to heat stress. The sharp decline in required exposure time between 55 °C and 65 °C is particularly noteworthy, as it defines an efficient and practical treatment window. This thermal sensitivity is likely driven by the acute denaturation of proteins and disruption of cellular membranes under high temperatures [48,49]. In contrast, achieving 99% mortality at −35 °C required substantially longer exposure, which may be attributed to the insect’s ability to synthesize cryoprotectants and its relatively high mean supercooling point [50], coupled with potentially enhanced cold adaptability when sheltered within phloem tissue. Our LET_99_ data provide robust, physiologically based parameters for designing reliable thermal disinfection protocols.

This study further revealed that wood severely infested by RHB often develops blue-stain fungus [51], necessitating its downgraded utilization. While the efficacy of beta-cypermethrin against adult beetles is well documented [52], we demonstrated that its fumigant formulation achieved mortality rates exceeding 99% against RHB in the bark and wood chip debris generated during processing of such low-value material. This high level of efficacy is particularly valuable for ensuring operational safety in populated environments such as wood-processing facilities. Furthermore, the finding that surviving insects were predominantly located in areas of thicker phloem indicates a limited ability of this insecticide to penetrate solid wood matrices.

Based on our empirical findings, we propose a novel, tiered management strategy that tailors treatment intensity to pest risk and wood value. For export or transport logs requiring absolute security, we recommend fumigation (selecting AP for deep penetration or SF for rapid treatment) or the validated high-temperature protocol (60–65 °C). For the downgraded utilization of wood within infested areas, mechanical debarking combined with beta-cypermethrin fumigation of residual debris offers a cost-effective solution, effectively achieving closed-loop management. Looking forward, while this study establishes a critical technical foundation, the scalability of these protocols—particularly fumigation uniformity in large, non-laboratory wood stacks—warrants further field-scale investigation. Future efforts should also monitor the potential development of resistance to beta-cypermethrin to ensure the long-term sustainability of this integrated approach.

## Figures and Tables

**Table 1:** Toxicity of AP and SF against RHB in 50 L sealed container at 20 °C.

Fumigant	Stage	LCT_50_ ^a^ (mg·h·L^−1^) (95% CL ^b^)	LCT_99_ (mg·h·L^−1^) (95% CL)	χ^2 e^	*df*	Slope ± SE	*p*-Value
AP ^c^	Egg	118.7 (111.0–127.0)	808.0 (681.1–958.6)	57.0	8	1.2 ± 0.1	<0.001
	Larva	82.9 (76.1–90.2)	623.8 (498.9–780.0)	37.6	5	1.2 ± 0.1	<0.001
	Pupa	105.0 (97.7–112.9)	732.5 (610.3–879.1)	51.6	7	1.2 ± 0.1	<0.001
	Adult	57.4 (52.0–63.5)	368.4 (300.5–451.7)	16.7	4	1.3 ± 0.1	<0.001
SF ^d^	Egg	41.8 (39.1–44.7)	204.7 (175.7–238.5)	38.9	6	1.5 ± 0.1	<0.001
	Larva	26.9 (24.8–29.2)	132.0 (112.2–155.2)	29.6	4	1.5 ± 0.1	<0.001
	Pupa	35.6 (33.2–38.3)	168.1 (143.8–196.4)	41.1	5	1.5 ± 0.1	<0.001
	Adult	22.2 (20.2–24.4)	132.4 (108.3–161.8)	26.4	3	1.3 ± 0.1	<0.001

^a^ LCT_50_ and _99_; 50% and 99% lethal concentration times. ^b^ Confidence limit. ^c^ Aluminum phosphide ^d^ Sulfuryl fluoride; ^e^ χ^2^, chi-square, from the Pearson goodness-of-fit test.

**Table 2:** Comparative Penetration of AP and SF in *Pinus thunbergii* wood.

Fumigant	Condition	LCT_50_ (mg·h·L^−1^) (95% CL)	LCT_99_ (mg·h·L^−1^) (95%CL)	χ^2^	*df*	Slope ± SE	*p*-Value	Penetration Rate (%)
AP	Open	97.8 (93.5–102.2)	279.4 (255.3–305.7)	8.4	8	2.2 ± 0.1	=0.401	29.5
	Sealed	215.6 (204.9–226.8)	948.7 (836.2–1076.2)	41.9	7	1.6 ± 0.1	<0.001	
SF	Open	25.3 (19.9–32.3)	81.4 (63.8–103.9)	0.3	3	2.0 ± 0.4	=0.969	12.6
	Sealed	160.8 (152.5–169.5)	644.8 (574.1–724.3)	12.7	8	1.7 ± 0.1	=0.121	

**Table 3:** Comparative Penetration of AP and SF in *P. thunbergii* wood stacks.

Fumigant	Depth	LCT_50_ (mg·h·L^−1^) (95% CL)	LCT_99_ (mg·h·L^−1^) (95%CL)	χ^2^	*df*	Slope ± SE	*p*-Value
AP	0.5	59.0 (54.6–63.8)	423.9 (342.8–524.1)	29.0	7	1.2 ± 0.1	<0.001
	1	59.5 (54.4–65.1)	603.5 (469.8–775.3)	21.4	7	1.0 ± 0.1	=0.003
	1.5	60.2 (54.9–66.1)	683.6 (537.3–869.7)	16.1	8	1.0 ± 0.1	=0.041
SF	0.5	22.9 (21.0–25.0)	130.5 (106.5–160.1)	18.5	5	1.3 ± 0.1	=0.002
	1	24.1 (22.2–26.2)	125.5 (103.4–152.4)	12.7	5	1.4 ± 0.1	=0.026
	1.5	27.8 (25.5–30.4)	177.0 (143.5–218.2)	5.9	5	1.3 ± 0.1	=0.318

**Table 4:** Impact of ambient temperature on the fumigation efficacy of SF and AP.

Fumigant	Temp. (℃)	LCT_50_ (mg·h·L^−1^) (95% CL)	LCT_99_ (mg·h·L^−1^) (95%CL)	χ^2^	*df*	Slope ± SE	*p*-Value
AP	11.5–15.4	889.3 (842.6–938.7)	8884.2 (7665.2–10,297.0)	114.4	18	1.0 ± 0.03	<0.001
	18.5–25.5	658.6 (620.7–698.8)	6421.7 (5584.2–7384.7)	178.9	18	1.0 ± 0.04	<0.001
	26.5–29.5	451.6 (414.0–492.5)	7373.1 (6136.3–8859.1)	200.9	16	0.8 ± 0.03	<0.001
SF	11.5–15.4	674.4 (635.3–715.8)	5146.5 (4502.7–5882.3)	110.5	15	1.1 ± 0.04	<0.001
	18.5–25.5	619.6 (583.2–658.3)	5101.0 (4394.8–5920.8)	99.6	14	1.1 ± 0.1	<0.001
	26.5–29.5	623.3 (584.9–664.3)	5795.5 (4945.2–6791.9)	60.6	15	1.0 ± 0.04	<0.001

**Table 5:** Impact of wood moisture content on the fumigation efficacy of SF and AP.

Fumigant	Moisture Content (%)	LCT_50_ (mg·h·L^−1^) (95% CL)	LCT_99_ (mg·h·L^−1^) (95%CL)	χ^2^	*df*	Slope ± SE	*p*-Value
AP	21.3–25.5	721.3 (329.8–826.0)	6264.6 (4712.1–8270.2)	38.9	5	1.1 ± 0.1	<0.001
	31.6–33.6	803.1 (706.4–913.0)	7278.1 (5401.1–9807.3)	49.6	5	1.1 ± 0.1	<0.001
	45.8–48.8	1055.0 (950.8–1170.6)	8649.2 (6634.1–11,276.4)	61.2	6	1.1 ± 0.1	<0.001
SF	21.3–25.5	456.9 (392.9–531.4)	6740.3 (4609.8–9855.4)	51.4	6	0.9 ± 0.1	<0.001
	31.6–33.6	532.1 (465.4–608.2)	7499.1 (5487.7–10,247.7)	45.9	8	0.9 ± 0.1	<0.001
	45.8–48.8	778.4 (705.3–859.0)	8510.9 (6522.0–11,106.3)	25.0	9	1.0 ± 0.1	<0.001

**Table 6:** Comparative efficacy of high and low temperatures in eradicating RHB from infested wood.

Thermal Treatment	Temp. (°C)	LET_50_ ^a^ (h) (95% CL)	LET_99_ (h) (95% CL)	χ^2^	*df*	Slope ± SE	*p*-Value
Heat	45	7.0 (6.1–8.0)	333.8 (233.4–477.3)	36.3	6	0.6 ± 0.04	<0.001
	50	5.0 (4.5–5.5)	37.5 (33.4–42.2)	71.2	6	1.2 ± 0.1	<0.001
	55	2.2 (1.8–2.8)	16.9 (14.5–19.2)	24.9	6	1.2 ± 0.1	<0.001
	60	1.9 (1.7–2.0)	7.0 (6.0–8.1)	0.86	3	1.8 ± 0.1	=0.835
	65	1.1 (0.9–1.3)	6.3 (5.3–7.4)	24.0	4	1.3 ± 0.1	<0.001
Cold	−15	383.5 (188.6–780.0)	69,417.9 (9553.0–504,435.8)	8.3	6	0.4 ± 0.1	=0.216
	−20	32.9 (30.8–35.3)	501.3 (364.3–689.8)	15.9	6	0.9 ± 0.04	=0.014
	−25	17.2 (16.3–18.1)	206.5 (168.9–252.4)	18.3	6	0.9 ± 0.04	=0.005
	−30	8.6 (8.2–9.0)	32.1 (29.6–34.8)	133.1	4	1.8 ± 0.1	<0.001
	−35	5.4 (5.1–5.7)	26.4 (24.2–28.8)	104.3	6	1.5 ± 0.1	<0.001

^a^ LET_50_ and _99_; 50% and 99% lethal exposure time.

## Data Availability

Data available in a publicly accessible repository: the data have been deposited in Zenodo with the following DOI: https://doi.org/10.5281/zenodo.17291707.

## References

[1] Yan Z., Sun J., Don O., Zhang Z. (2005). The red turpentine beetle, *Dendroctonus valens* LeConte (Scolytidae): An exotic invasive pest of pine in China. Biodivers. Conserv..

[2] Santini A., Faccoli M. (2014). Dutch elm disease and elm bark beetles: A century of association. Iforest-BiogeoSci. For..

[3] Lin W., Park S., Jiang Z.-R., Ji Y.-C., Ernstsons A.S., Li J.-J., Li Y., Hulcr J. (2021). Native or Invasive? The Red-Haired Pine Bark Beetle *Hylurgus ligniperda* (Fabricius) (Curculionidae: Scolytinae) in East Asia. Forests.

[4] Faccoli M., Gallego D., Branco M., Brockerhoff E.G., Corley J., Coyle D.R., Hurley B.P., Jactel H., Lakatos F., Lantschner V. (2020). A first worldwide multispecies survey of invasive Mediterranean pine bark beetles (Coleoptera: Curculionidae, Scolytinae). Biol. Invasions.

[5] Mausel D.L., Gara R.I., Lanfranco D., Ruiz C., Ide S., Azat R. (2007). The introduced bark beetles *Hylurgus ligniperda* and *Hylastes ater* (Coleoptera: Scolytidae) in Chile: Seasonal flight and effect of *Pinus radiata* log placement on colonization. Can. J. For. Res..

[6] Chase K.D., Kelly D., Liebhold A.M., Bader M.K.-F., Brockerhoff E.G. (2017). Long-distance dispersal of non-native pine bark beetles from host resources. Ecol. Entomol..

[7] Clare G., George E.M. (2016). Life cycle and mass-rearing of *Hylurgus ligniperda* using a novel egg-collection method. New Zealand Plant Prot..

[8] Park S., Jung J., Han T. (2017). A new species and five newly recorded species of Scolytinae (Coloptera: Curculionidae) from Korea. Entomol. Res. Bull..

[9] Ren L.L., Tao J., Wu H.W., Zong S.X., Wang C., Hua D., Shi J., Liu Y.Z., Luo Y.Q. (2021). The first discovery and infective characteristics of a major invasive pest *Hylurgus ligniperda* (Coleoptera: Scolytidae) in China. Sci. Silvae Sin..

[10] Hoebeke E. (2001). *Hylurgus ligniperda*: A new exotic pine bark beetle in the United States. Newsl. Mich. Entomol. Soc..

[11] Jankowiak R., Bilański P. (2013). Ophiostomatoid fungi associated with root-feeding bark beetles on Scots pine in Poland. For. Pathol..

[12] de Errasti A., Pildain M.B., Rajchenberg M. (2018). Ophiostomatoid fungi isolated from three different pine species in Argentinian Patagonia. For. Pathol..

[13] Lu M., Hulcr J., Sun J. (2016). The role of symbiotic microbes in insect invasions. Annu. Rev. Ecol. Evol. Syst..

[14] Fabre J.-P., Carle P. (1975). Contribution à l’étude biologique d’*Hylurgus Ligniperda* F. (Coleoptera Scolytidae) dans le Sud-est de la France. Ann. Des Sci. For..

[15] Page A.B.P., Lubatti O.F. (1963). Fumigation of insects. Annu. Rev. Entomol..

[16] Mellouki A., Talukdar R.K., Schmoltner A.-M., Gierczak T., Mills M.J., Solomon S., Ravishankara A.R. (1992). Atmospheric lifetimes and ozone depletion potentials of methyl bromide (CH3Br) and dibromomethane (CH2Br2). Geophys. Res. Lett..

[17] Najar-Rodriguez A.J., Hall M.K.D., Adlam A.R., Afsar S., Esfandi K., Wilks C., Noakes E., Clare G.K., Barrington A., Brash D.W. (2020). Efficacy of quarantine treatments using reduced methyl bromide concentrations to disinfest *Pinus radiata* logs from New Zealand. J. Stored Prod. Res..

[18] Porter I., Banks J., Mattner S., Fraser P., Gisi U., Chet I., Gullino M.L. (2009). Global phaseout of methyl bromide under the montreal protocol: Implications for bioprotection, biosecurity and the ozone layer. Recent Developments in Management of Plant Diseases.

[19] Sankarganesh E., Girish A.G., Alice R.P., Mariadoss A. (2020). Influence of relative humidity on phosphine concentration during aluminium phosphide (ALP) fumigation in pigeon pea. Int. J. Chem. Stud..

[20] Najar-Rodriguez A.J., Afsar S., Esfandi K., Hall M.K.D., Adlam A.R., Wilks C., Noakes E., Richards K. (2020). Laboratory toxicity and large-scale commercial validation of the efficacy of ethanedinitrile, a potential alternative fumigant to methyl bromide, to disinfest New Zealand Pinus radiata export logs. J. Stored Prod. Res..

[21] Abrams A.E., Kawagoe J.C., Najar-Rodriguez A., Walse S.S. (2020). Sulfuryl fluoride fumigation to control brown marmorated stinkbug (Hempitera: Pentatomidae). Postharvest Biol. Technol..

[22] Lee B.-H., Yang J.-O., Beckett S., Ren Y. (2017). Preliminary trials of the ethanedinitrile fumigation of logs for eradication of *Bursaphelenchus xylophilus* and its vector insect *Monochamus alternatus*. Pest Manag. Sci..

[23] Spence D.J., Smith J.A., Ploetz R., Hulcr J., Stelinski L.L. (2013). Effect of chipping on emergence of the redbay ambrosia beetle (Coleoptera: Curculionidae: Scolytinae) and recovery of the laurel wilt pathogen from infested wood chips. J. Econ. Entomol..

[24] Pawson S.M., Bader M.K.F., Brockerhoff E.G., Heffernan W.J.B., Kerr J.L., O’Connor B. (2019). Quantifying the thermal tolerance of wood borers and bark beetles for the development of Joule heating as a novel phytosanitary treatment of pine logs. J. Pest Sci..

[25] Eliopoulos P.A., Prasodimou G.Z., Pouliou A.V. (2011). Time–mortality relationships of larvae and adults of grain beetles exposed to extreme cold. Crop Prot..

[26] Estes P.M. (1965). The effects of time and temperature on methyl bromide fumigation of adults of *Sitophilus granarius* and *Tribolium confusum*. J. Econ. Entomol..

[27] Nayak M.K., Collins P.J. (2008). Influence of concentration, temperature and humidity on the toxicity of phosphine to the strongly phosphine-resistant psocid *Liposcelis bostrychophila* Badonnel (Psocoptera: Liposcelididae). Pest Manag. Sci..

[28] Park C.G., Son J.K., Lee B.H., Cho J.H., Ren Y. (2014). Comparison of ethanedinitrile (C2N2) and metam sodium for control of *Bursaphelenchus xylophilus* (Nematoda: Aphelenchidae) and *Monochamus alternatus* (Coleoptera: Cerambycidae) in naturally infested logs at low temperatures. J. Econ. Entomol..

[29] Zhao L., Ramaswamy H., Wang S. (2018). Thermal-death kinetics of the bark beetle (*Dendroctonus armandi*; Coleoptera: Scolytidae). Scand. J. For. Res..

[30] Noseworthy M.K., Humble L.M., Souque T.J., John E.P., Roberts J., Lloyd C.R., Allen E.A. (2023). Determination of specific lethal heat treatment parameters for pests associated with wood products using the Humble water bath. J. Pest Sci..

[31] Hopf-Biziks A., Schröder T., Schütz S. (2017). Long-term survival and non-vector spread of the pinewood nematode, *Bursaphelenchus xylophilus*, via wood chips. For. Pathol..

[32] Gu Y., Ge S., Li J., Ren L., Wang C., Luo Y. (2024). Composition and diversity of the endobacteria and ectobacteria of the invasive bark beetle *Hylurgus ligniperda* (Fabricius) (Curculionidae: Scolytinae) in newly colonized areas. Insects.

[33] Romo C.M., Bader M.K., Pawson S.M. (2016). Comparative growth and survival of *Hylurgus ligniperda* (Coleoptera: Scolytinae) and *Arhopalus ferus* (Coleoptera: Cerambycidae) reared on artificial or natural diet at 15 or 25 °C. J. Econ. Entomol..

[34] Throne J., Weaver D., Chew V., Baker J. (1995). Probit Analysis of Correlated Data: Multiple observations over time at one concentration. J. Econ. Entomol..

[35] Brockerhoff E.G., Schläfli L., Cornejo C., Kappeler J., Orbach J., Tiefenbacher A., Kupper Q., Avtzis D., Branco M., Carnegie A.J. (2025). Worldwide spread of *Hylurgus ligniperda* (Coleoptera: Scolytinae), and the potential role of bridgehead invasions. bioRxiv.

[36] Pranamornkith T., Hall M., Adlam A., Page B., Connolly P., Somerfield K., Brash D. (2014). Relative methyl bromide tolerances of *Arhopalus ferus* (Mulsant), *Hylurgus ligniperda* (F.) and *Hylastes ater* (Paykull) adults. New Zealand Plant Prot..

[37] Brockerhoff E., Bain J., Kimberley M., Knizek M. (2006). Interception frequency of exotic bark and ambrosia beetles (Coleoptera: Scolytinae) and relationship with establishment in New Zealand and worldwide. Can. J. For. Res..

[38] Ide S., Valenzuela J., Estay S., Jaksic F., Castro S. (2014). Presión de ingreso de insectos forestales exóticos a Chile desde 1996. En libro invasiones biológicas en chile: Causas globales e impactos locales. Invasiones Biológicas en Chile: Causas Globales e Impactos Locales.

[39] Turner R.M., Liebhold A.M., Nahrung H.F., Phillips C.B., Yamanaka T., Brockerhoff E.G. (2024). The known unknowns in international border interceptions of non-native insects. Biol. Invasions.

[40] Yan D., Liu J., Wang X., Fang W., Li Y., Cao A., Wang Q. (2025). A review on the mechanisms of fumigant action. New Plant Prot..

[41] Jeon J.C., Kim H.K., Koo H.N., Kim B.S., Yang J.O., Kim G.H. (2022). Synergistic Effect of cold treatment combined with ethyl formate fumigation against *Drosophila suzukii* (Diptera: Drosophilidae). Insects.

[42] Ramadan G.R.M., Mosallam E.M., Phillips T.W. (2024). Methyl benzoate and its derivative, acetophenone, as fumigants to control stored product insects. J. Stored Prod. Res..

[43] Jagadeesan R., Singarayan V.T., Nayak M.K. (2021). A co-fumigation strategy utilizing reduced rates of phosphine (PH3) and sulfuryl fluoride (SF) to control strongly resistant rusty grain beetle, *Cryptolestes ferrugineus* (Stephens) (Coleoptera: Laemophloeidae). Pest Manag. Sci..

[44] Su N.-Y., Scheffrahn R.H. (2014). Efficacy of sulfuryl fluoride against four beetle pests of museums (Coleoptera: Dermestidae, Anobiidae). J. Econ. Entomol..

[45] Eisenback J.D., Chen Z., White M. (2024). Evaluating vacuum and steam heat to eliminate pinewood nematodes in naturally infested whole pine logs. J. Nematol..

[46] Zou H., Li L., Zhang J., Li B., Xiao Y., Ren Y., Huang J., Chen W., Liu T. (2025). Low-temperature phosphine fumigation is effective against *Drosophila suzukii* in sweet cherry. Insects.

[47] Kim M.J., Kim J.S., Jeong J.S., Choi D.S., Park J., Kim I. (2018). Phytosanitary cold treatment of spotted-wing *Drosophila*, *Drosophila suzukii* (Diptera: Drosophilidae) in ‘campbell early’ grape. J. Econ. Entomol..

[48] King A.M., MacRae T.H. (2015). Insect heat shock proteins during stress and diapause. Annu. Rev. Entomol..

[49] Cooper B.S., Hammad L.A., Montooth K.L. (2014). Thermal adaptation of cellular membranes in natural populations of *Drosophila melanogaster*. Funct. Ecol..

[50] Cheng L., Pei J., Chen X., Shi F., Bao Z., Hou Q., Zhi L., Zong S., Tao J. (2024). Cold tolerance and metabolism of red-haired pine bark beetle *Hylurgus ligniperda* (Coleoptera: Curculionidae) during the overwintering period. J. Econ. Entomol..

[51] Hýsek Š., Löwe R., Turčáni M. (2021). What happens to wood after a tree is attacked by a bark beetle?. Forests.

[52] Chen R., Zhou J., Zhang H., Xing Y., Chi D., Yu J. (2025). Breaking the beetle barrier: Innovative strategies for controlling *Hylurgus ligniperda* in China. Ecotoxicol. Environ. Saf..

